# Parent experiences with genetic testing for pediatric hearing loss

**DOI:** 10.1002/jgc4.1986

**Published:** 2024-11-05

**Authors:** Ivette Cejas, Jennifer Coto, Christina M. Sarangoulis, Valerie Yunis, Susan Blanton, Xue Zhong Liu

**Affiliations:** ^1^ Department of Otolaryngology University of Miami Miller School of Medicine Miami Florida USA; ^2^ Department of Pediatrics University of Miami Miller School of Medicine Miami Florida USA; ^3^ John P. Hussman Institute for Human Genomics, John T. Macdonald Foundation Department of Human Genetics University of Miami Miller School of Medicine Miami Florida USA

**Keywords:** barriers, caregiver, genetic counseling, genetic evaluation, pediatric hearing loss

## Abstract

The purpose of the current study was to assess parent perceptions and experiences of genetic testing, as well as barriers for not undergoing testing in a sample of families of children with hearing loss. A 44‐item questionnaire, The Parent Perception of Genetic Testing Questionnaire developed by the research study team was administered. Participants were recruited from a pediatric otolaryngology/audiology practice and social media. A total of 146 parents of children with hearing loss participated. Approximately 47.6% of the children in our sample underwent genetic testing, 44.8% did not, and 7.6% of parents were unsure. For those that did not undergo testing, reasons included: unaware (6.2%), not interested (8.9%), cost (8.9%), time (3.4%), fearful of results (2.7%), and never offered (15.1%). For those that did undergo testing, over half of the parents reported that they did not receive counseling before (55.1%) and 41.7% reported they received counseling after the testing. Furthermore, parents were confused about the results with 18.3% reporting they were Very Confused, 28.3% Somewhat Confused, 20% A Little Confused, and 33.3% Not Confused at All about the variant of uncertain or unknown significance. Notably, less than half of parents (43.4%) remembered what they were told about the mode of inheritance. Overall, our study highlighted the low adoption rate of genetic testing and lack of integration into standard of care for otology/audiology practices. Collaboration between hearing healthcare professionals and geneticists is warranted to determine how to reduce barriers to access while improving pre‐ and post‐counseling.

**What is known about this topic**

Genetic evaluation can support treatment planning for individuals with hearing loss, as well as support counseling related to prognosis and speech and language development. Issues related to access to geneticists and reimbursement have been reported as barriers for undergoing genetic evaluation.

**What this paper adds to the topic**

This study highlights the lack of integration of genetic testing as part of otolaryngology and audiology practices management of pediatric hearing loss. In addition, it showed that more work is needed to improve the pre‐ and post‐counseling following genetic evaluation for this population.

## 1 INTRODUCTION

Genomic and precision medicine have rapidly evolved over the past decade and have the potential to improve the diagnosis and treatment of sensory disorders, including deafness. According to recommendations from the American College of Medical Genetics and Genomics (ACMG) and an International Pediatric Otolaryngology consensus group, all children with bilateral sensorineural hearing loss should undergo a comprehensive genetic evaluation, including patient‐focused medical and birth histories, physical exam and family history (Li et al., 2022; Liming et al., 2016). The diagnostic rate is estimated to be 40%–65%, depending on ethnicity and severity of hearing loss. Recent research has attributed over 100 genes to be associated with non‐syndromic sensorineural hearing loss (Davis et al., 2021). Common mutations observed in children include those targeting *GJB2*, *SLC26A4, MYO7A*, *CDH23*, *LOXHD1*, *ACXTG1*, *COCH*, *ROR1*, *OPA1*, among others (Nisenbaum et al., 2021). Despite the evidence supporting genetic testing, it has not been fully adopted within otolaryngology and audiology practices.

In fact, while the American College of Medical Genetics released a guideline in 2014 that incorporated comprehensive genetic testing as part of standard of care; research has shown that integration into clinical settings has been a slow process. Jayawardena et al. (2015) reported that only 37% of otolaryngologists ordered genetic testing at their first patient encounter and this dropped to 30% in their second patient visit. Another study reported that only 35% of patients received a genetic referral with authors concluding that medical care for children with hearing loss is variable and inconsistent across centers (Judge et al., 2019). Consistent with these studies, the literature has documented that less than half of families and patients with hearing loss actually pursue genetic testing (Lesperance et al., 2018). The identification of genes can aid in individual treatment planning and determining prognosis and outcomes. Awareness of etiology can also help in decision‐making for hearing aids or cochlear implantation and in some cases can prevent a delay in decision‐making to move forward with hearing rehabilitation (Kim & Choi, 2022; Withrow et al., 2009). Recent work has also reported that outcomes for speech perception may also vary by genotype for individuals using cochlear implants (Carlson et al., 2023).

While genetic testing is known to be beneficial to the medical community and health providers, there continues to be challenges to equal access to these services for diverse communities. In a focus group study with adults and parents of children with hearing loss, participants reported several intrinsic and extrinsic barriers for obtaining genetic testing (Lesperance et al., 2018). Specifically, individuals reported difficulty obtaining referrals, scheduling issues, financial concerns related to insurance coverage, and lack of awareness about the utility of diagnostic testing (Lesperance et al., 2018). This study also highlighted the less than optimal access that individuals had to genetic testing, suggesting that genetic clinics may prioritize more complex medical cases thus reducing opportunities for patients with only hearing loss. This in addition to being overwhelmed with the initial diagnosis often diminishes the importance of seeking genetic testing.

To date, the majority of research regarding patient attitudes towards genetic testing in hearing loss has been conducted by Withrow et al. (2009). In one study, they conducted two separate surveys to analyze parental attitudes towards genetic testing for individuals who identified as being part of the deaf and/or hearing community. Interestingly, only 3.1% of parents whose children had undergone genetic testing reported that it was due to their physician recommendation/referral. This study also found that most participants who either identified with the Hearing community or had previously undergone genetic testing reported more positive feelings towards genetic testing. Those individuals who had pursued genetic testing described stronger beliefs in genetic causes for hearing loss. Overall, parents who identified as being part of the hearing community were interested in determining the cause of their child's hearing loss.

Withrow et al. (2009) also addressed the changes in the perspectives of the deaf community and genetic testing. Historically, the deaf community has had more negative attitudes towards testing, however, Withrow and colleagues reported that these perspectives are malleable and more work is needed related to educating the larger community on genetic testing and the benefits, which would result in greater acceptance. In this study, individuals identifying as part of the deaf community were less likely to pursue genetic testing and their motivation for seeking testing was mostly related to providing their family with more information or determining chances of reoccurrence.

Given the increase in access to genetic testing and improved insurance coverage over the past few years, the current study sought to examine whether barriers to genetic testing in pediatric hearing loss continue and whether there has been an increase in the uptake of genetic testing as part of hearing loss evaluation and management. Our first aim was to assess parent perceptions of genetic testing and parent experiences with either seeking or undergoing evaluation in a sample of families of children with confirmed hearing loss. Further, for those who did not pursue testing, we sought to identify the reasons and/or barriers for not undergoing testing. Obtaining clarification for the persistent barriers that affect access to genetic testing is the first step towards integrating precision medicine in otolaryngology and audiology practices. We expected that less than half of our sample will have undergone genetic testing. We did not expect any differences in the proportion of individuals pursuing genetic testing by basic demographic variables (i.e., parent age, race, ethnicity). Further, we expected that insurance coverage would not be the sole barrier to undergoing genetic testing. More specifically in accordance with the current literature we anticipated that parents would primarily report unaware of genetic testing benefits or never referred as the primary reasons for not pursuing testing.

## 2 MATERIALS AND METHODS

Parents were recruited from a pediatric otolaryngology/audiology practice at an academic medical center and social media hearing loss groups. The otolaryngology/audiology practice includes a multidisciplinary team of audiologists, otologists, speech and language pathologists, psychologists, educator, and social worker. The team also includes a geneticist, who runs a clinic once a week at the same location. An email invitation was sent to parents of children with hearing loss in the clinic and if interested, parents were provided with the link to the online survey. The survey was also posted on social media groups for parents of children with hearing loss, including the following Facebook groups: Parents of children with cochlear implants and Support group for parents of kids with hearing loss. The flyer that was posted had a QR code and link which participants could select or scan if they were interested in participating. The online survey was provided in English and was active for approximately 1 month in February 2022. Inclusion criteria consisted of parents of children with hearing loss who read/understood English. No families were excluded from participation. All parents of children with hearing loss, regardless of degree or etiology of hearing loss were included (e.g., unilateral, bilateral, sensorineural, conductive). Following electronic consent, parents completed the online survey assessing their perceptions, decisions, and satisfaction with genetic testing that was administered via Qualtrics. This platform was used as it is a user‐friendly interface that has built in security measures to prevent spam responses. This study protocol was reviewed and approved by the University of Miami Institutional Review Board, protocol number 20190540.

### 2.1 Measures

#### 2.1.1 Demographics questionnaire

The demographic questionnaire included questions regarding both the parent and child. Parent demographics included: relationship to the child, age, race, ethnicity, highest level of educational attainment, occupation, income, and age of other children in the household. Child demographics included age, race, ethnicity, and gender.

#### 2.1.2 Parent perception of genetic testing questionnaire

The Parent Perception of Genetic Testing Questionnaire was developed by the research study team who included experts in genetics of hearing loss (i.e., geneticist, researcher, psychologist). It was adapted from the questionnaire created by Withrow and colleagues, who were consultants on the grant that funded this work (Withrow et al., 2009). The PPGTQ is a non‐standardized questionnaire that included 44 items that assessed family history of hearing loss, awareness of genetic testing and newborn screening, perceptions of genetic testing and family planning (see the Appendix S1). The PPGTQ also includes questions related to the onset, etiology and severity of hearing loss, as well as use of hearing technology. For those who underwent genetic testing, questions were then asked about the results of testing such as if a gene was identified, if the family received any counseling before and/or after genetic testing and decisions on who the information was shared with. If the child did not receive genetic testing, questions were asked about the reason for not pursing genetic testing and current interest in genetic testing. Participants were instructed to select one response per item, except for the question related to parents' feelings regarding new discoveries for genetics in hearing.

### 2.2 Data analysis plan

Analyses for the electronic survey were conducted using the Statistical Package for Social Sciences, version 28 (IBMCorp, 2021). All complete responses were analyzed (*n* = 146). Descriptive analyses were conducted for all demographic variables and all survey responses (means, frequencies). To examine whether there were any fundamental differences in our sample by certain variables (genetic testing status; recruitment approach, insurance coverage), we conducted correlations and follow‐up chi‐squared tests across demographic and audiology variables (i.e., ethnicity, race, insurance coverage). First, the sample was stratified into those who underwent genetic testing and those who did not. Second, to assess whether our recruitment approach impacted perspectives on genetic testing, we separated our sample by those who were recruited from clinic versus social media. Finally, to examine the impact of insurance, the sample was divided into those who had coverage for genetic testing and those who did not. Data for the PPGTQ responses were analyzed for the entire sample, as no major group differences across genetic testing status, recruitment approach, or insurance coverage were noted across the key demographic and audiological variables. As is noted in the PPGTQ data (see the Appendix S1), questions related to reasons not to pursue testing were only asked for those who did not undergo testing. Similarly, certain questions regarding genetic counseling were only asked for those who underwent testing and thus will only be presented for this subgroup.

## 3 RESULTS

### 3.1 Parent and child demographics

A total of 146 parents participated in the study. Ninety‐two were recruited from social media and 54 from a comprehensive pediatric practice. Most parents were mothers (91.8%), between the ages of 30–49 years (49.3%), and identified as non‐Hispanic (76.6%) and White (93.1%). Most parents completed college or earned a graduate degree (55.5%) and 75.4% had a household income above $50,000 per year. Eleven percent of the parents reported being deaf or hard of hearing themselves with 75% attributing their hearing loss to a genetic condition. Children had a mean age of 6.58 (SD = 5.33) years and 45.5% were female. Most parents identified their child as White (90.4%) and non‐Hispanic (74.7%). The majority of children had congenital hearing loss (63.1%) and most had a profound hearing loss in their worst ear. Most of the sample did not know the cause of their child's hearing loss (47.3%), followed by probably genetic cause (40.4%), infectious diseases (5.5%), and other (6.8%). Twenty‐five percent of the children wore cochlear implants, 66.4% wore hearing aids, 5.5% wore a bone conduction device, and 6.8% were unaided (See Table 1 for further descriptive information).

**TABLE 1:** Parent and child demographic and audiological information.

*n* = 146	M (SD)	*N* (%)
Child age (years)	6.58 (5.33)	
Child sex (female)		66 (45.5%)
Child ethnicity (Hispanic)		37 (25.3%)
Child race		
Asian		3 (2.1%)
Black/African American		10 (6.8%)
White		132 (90.4%)
Other		1 (0.7%)
Child hearing device		
Cochlear implant		37 (25.3%)
Hearing aid		97 (66.4%)
Bone conduction device		8 (5.5%)
Unaided		10 (6.8%)
Onset of child hearing loss		
Congenital		82 (63.10%)
Progressive		27 (20.8%)
Sudden		21 (16.2%)
Child health insurance type		
Private		88 (60.3%)
Public		43 (29.5%)
Military		9 (6.2%)
No insurance		1 (0.7%)
Child cultural identification (parent report)		
Deaf		4 (2.7%)
Hearing		123 (84.2%)
Deaf and hearing equally		19 (13%)
Relationship to child (mother)		134 (91.8%)
Parent age		
20–29 years		21 (14.4%)
30–39 years		72 (49.3%)
40–49 years		44 (30.1%)
50–59 years		6 (4.1%)
60 years+		3 (2.1%)
Parent hearing status		
Deaf		2 (1.4%)
Hard of hearing		14 (9.6%)
Hearing		130 (89%)
Parent cultural identification		
Deaf		1 (0.7%)
Hearing		130 (89%)
Deaf and hearing equally		12 (8.2%)
Other		3 (2.1%)
Parent highest education		
Some high school or less		5 (3.5%)
High school		13 (8.9%)
Some college/2‐year college		38 (30.2%)
4‐year University		40 (31.7%)
Graduate school or higher		30 (23.8%)

### 3.2 Effects of demographic variables on rates of genetic testing

Child onset of hearing loss (*r*_*s*_ = −0.18, *p* < 0.05) and parent highest level of education (*r*_*s*_ = −0.19, *p* < 0.05) were significantly correlated with undergoing genetic testing. However, ad hoc sample proportion tests revealed no significant differences in proportions between any of the groups, respectively (see Table 2). As expected, there were no differences in whether children underwent genetic testing (Hispanic: 50%, Non‐Hispanic: 46.8%; *p* = 0.96, *r*_*s*_ = 0.004) or not based on ethnicity.

**TABLE 2:** Differences in undergoing genetic testing by demographic and audiological variables.

	Had genetic testing (*n* = 69)	No genetic testing (*n* = 65)	Significant correlations	Chi‐square
Child age in years—M (SD)	5.74 (4.84)	7.05 (5.80)		
Child sex (female)	29 (42.6%)	31 (47.7%)		
Child ethnicity (Hispanic)	18 (26.1%)	14 (21.5%)		
Child race				
Asian	1 (1.4%)	2 (3.1%)		
Black/African American	3 (4.3%)	5 (7.7%)		
White	64 (92.8%)	58 (89.2%)		
Other	1 (1.4%)	0 (0%)		
Child hearing device				
Cochlear implant	22 (31.9%)	12 (18.5%)		
Hearing aid	44 (63.8%)	47 (72.3%)		
Bone conduction device	3 (4.3%)	4 (6.2%)		
Unaided	5 (7.2%)	4 (6.2%)		
Onset of child hearing loss			*r*_*s*_ = −0.18, *p* < 0.05	*χ* ^2^ = 2.46, *p* = 0.293
Congenital	44 (71.0%)	34 (57.6%)		
Progressive	11 (17.7%)	14 (23.7%)		
Sudden	7 (11.3%)	11 (18.6%)		
Child health insurance type				
Private	45 (65.2%)	41 (63.1%)		
Public	17 (24.6%)	19 (29.2%)		
Military	5 (7.2%)	3 (4.6%)		
No insurance	0 (0%)	1 (1.5%)		
Child cultural identification (parent report)				
Deaf	0 (0%)	2 (3.1%)		
Hearing	60 (87.0%)	54 (83.1%)		
Deaf and hearing equally	9 (13.0%)	9 (13.8%)		
Relationship to child (mother)	62 (89.9%)	62 (95.4%)		
Parent age				
20–29 years	9 (13.0%)	9 (13.8%)		
30–39 years	35 (50.7%)	31 (47.7%)		
40–49 years	20 (29.0%)	22 (33.8%)		
50–59 years	5 (7.2%)	0 (0%)		
60 years+	0 (0%)	3 (4.6%)		
Parent hearing status				
Deaf	0 (0%)	1 (1.5%)		
Hard of hearing	5 (7.2%)	8 (12.3%)		
Hearing	64 (92.8%)	56 (86.2%)		
Parent cultural identification				
Deaf	0 (0%)	0 (0%)		
Hearing	61 (88.4%)	59 (90.8%)		
Deaf and hearing equally	6 (8.7%)	6 (9.2%)		
Other	2 (2.9%)	0 (0%)		
Parent highest education			*r*_*s*_ = −0.19, *p* < 0.05	*χ* ^2^ = 0.57, *p* = 0.967
Some high school or less	2 (3.3%)	1 (1.9%)		
High school	5 (8.2%)	5 (9.3%)		
Some college/2‐year college	17 (27.9%)	15 (27.8%)		
4‐year University	20 (32.8%)	20 (37.0%)		
Graduate school or higher	17 (27.9%)	13 (24.1%)		

*Note*: Eight participants indicated they were not sure if their child had undergone genetic testing and therefore are not included in this table.

### 3.3 Social media versus clinic sample

Child age (*r*_*s*_ = −0.42, *p* ≤ 0.001), child ethnicity (*r*_*s*_ = 0.40, *p* < 0.001), health insurance type (*r*_*s*_ = −0.18, *p* < 0.05), relationship to child (*r*_*s*_ = −0.29, *p* < 0.001) and parent age (*r*_*s*_ = −0.29, *p* < 0.001) were all significantly correlated with recruitment approach (social media versus clinic). Specifically, there was a higher proportion of Hispanic respondents recruited from clinic (48.1%) as compared to those recruited from social media (12%; chi‐square = 21.68, *p* < 0.001), as well as a higher proportion of individuals with private insurance in the social media group (48.1% vs. 67.4%; chi‐square = 4.49, *p* < 0.05). In contrast, there was a higher proportion of individuals with public insurance recruited from clinic (44.4% vs. 20.7%, chi‐square = 8.16, *p* < 0.05). No differences were found between groups for those with military insurance (3.7% vs. 7.6%; chi‐square = 0.35, *p* = 0.56). As expected, there was a statistically significant higher proportion of mothers in the social media group (97.8%) compared to the clinic group (81.5%); (chi‐square = 9.98, *p* < 0.05); however, both samples primarily included mothers as respondents. Lastly, there were statistically significant differences in parent age between groups, with more 30–39‐year‐olds in the social media group (57.6% vs. 35.2%; chi‐square = 5.98, *p* < 0.01) compared to the clinic group. All other age groups were not significantly different (20–29 chi‐square = 0.38, *p* = 0.54; 40–49 chi‐square = 1.45, *p* = 0.23; see Table 3).

**TABLE 3:** Comparison of the clinic versus social media sample.

	Clinic (*n* = 54)	Social media (*n* = 92)	Significant correlations	Chi‐square
Child age in years—M (SD)	9.69 (5.91)	4.80 (4.03)	*r*_*s*_ = −0.42, *p* < 0.001	
Child sex (female)	21 (39.6%)	45 (48.9%)		
Child ethnicity (Hispanic)	26 (48.1%)	11 (12.0%)	*r*_*s*_ = 0.40, *p* < 0.001	*χ* ^2^ = 21.68, *p* < 0.001^a^
Child race				
Asian	0 (0%)	3 (3.3%)		
Black/African American	5 (9.3%)	5 (5.4%)		
White	49 (90.7%)	83 (90.2%)		
Other	0 (0%)	1 (1.1%)		
Child hearing device				
Cochlear implant	26 (48.1%)	11 (12.0%)		
Hearing aid	24 (44.4%)	73 (79.3%)		
Bone conduction device	1 (1.9%)	7 (7.6%)		
Unaided	5 (9.3%)	5 (5.4%)		
Onset of child hearing loss				
Congenital	29 (64.4%)	53 (62.4%)		
Progressive	8 (17.8%)	19 (22.4%)		
Sudden	8 (17.8%)	13 (15.3%)		
Child health insurance type				*r*_*s*_ = −0.18, *p* < 0.05
Private	26 (48.1%)	62 (67.4%)		*χ* ^2^ = 4.49, *p* < 0.05^a^
Public	24 (44.4%)	19 (20.7%)		*χ* ^2^ = 0.35, *p* < 0.56
Military	2 (3.7%)	7 (7.6%)		*χ* ^2^ = 0.35, *p* = 0.56
No insurance	1 (1.9%)	0 (0%)		
Child cultural identification (parent report)				
Deaf	2 (3.7%)	2 (2.2%)		
Hearing	46 (85.2%)	77 (83.7%)		
Deaf and hearing equally	6 (11.1%)	13 (14.1%)		
Relationship to child (Mother)	44 (81.5%)	90 (97.8%)	*r*_*s*_ = −0.29, *p* < 0.001	*χ* ^2^ = 9.98, *p* < 0.05^a^
Parent age				*r*_*s*_ = −0.29, *p* < 0.001
20–29 years	6 (11.1%)	15 (16.3%)		*χ* ^2^ = 0.38, *p* = 0.54
30–39 years	19 (35.2%)	53 (57.6%)		*χ* ^2^ = 5.98, *p* = 0.01^a^
40–49 years	20 (37%)	24 (26.1%)		*χ* ^2^ = 1.45, *p* = 0.23
50–59 years	6 (11.1%)	0 (0%)		–
60 years+	3 (5.6%)	0 (0%)		
Parent hearing status				
Deaf	1 (1.9%)	1 (1.1%)		
Hard of hearing	2 (3.7%)	12 (13.0%)		
Hearing	51 (94.4%)	79 (85.9%)		
Parent cultural identification				
Deaf	0 (0%)	1 (1.1%)		
Hearing	46 (85.2%)	84 (91.3%)		
Deaf and hearing equally	6 (11.1%)	6 (6.5%)		
Other	2 (3.7%)	1 (1.1%)		
Parent highest education				
Some high school or less	1 (2.4%)	4 (4.8%)		
High school	7 (16.7%)	6 (7.1%)		
Some college/2‐year college	8 (19.0%)	30 (35.7%)		
4‐year University	14 (33.3%)	26 (31.0%)		
Graduate school or higher	12 (28.6%)	18 (21.4%)		

^a^ Significant difference in proportions between groups.

### 3.4 Insurance

Over half of the children (60.3%) had private insurance, with 43.8% of parents reporting that their child's health plan included some coverage for genetic testing, 12.3% reported that it did not, and 43.8% were unsure. Thus, our data suggest that insurance coverage may not directly affect a family's decision to undergo genetic testing as suggested by similar rates for those with versus without coverage (no coverage: 55.6%; coverage: 62.5%; *p* = 0.72, *r*_*s*_ = 0.040). This supports our hypothesis that insurance coverage may not be the primary or sole reason why individuals do not seek a comprehensive genetic evaluation.

### 3.5 Parent perceptions of genetic testing

Results from the survey for the entire sample revealed that nearly half of the parents who participated were unsure of the etiology of their child's hearing loss (43.8%). Approximately 47.6% of the children in our sample underwent genetic testing, 44.8% did not, and 7.6% of parents were unsure. Overall, parents reported a range of positive feelings when asked how they felt about new discoveries in the genetics of hearing (e.g., excited 64.4%, hopeful 41.1%, enthusiastic 28.1%, positive 45.9%). However, 19.9% of parents also reported mixed feelings, including feelings of confusion (4.1%), worry (5.5%), cautiousness (9.6%) or concerns (1.4%). Most parents reported that it is somewhat to very important (91%) that someone who is fluent in their language discuss genetic testing with them, while 7.6% were neutral and 1.4% reported that it was not important. The full sample was also asked who they preferred to talk to about the results of genetic testing regardless of whether they had obtained testing or not. Sixty‐three percent of parents preferred a geneticist, 64.4% a genetic counselor, 52.1% audiologist, 37.7% ENT physician, 25.3% and pediatrician/family doctor.

#### 3.5.1 Non‐genetic testing group

**FIGURE 1:**
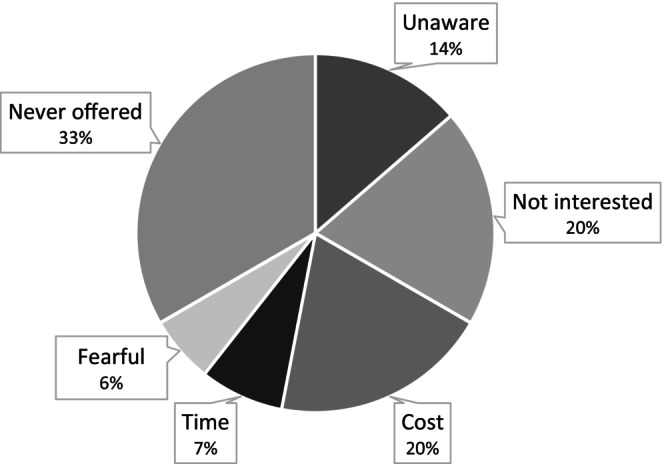
Top reasons for not undergoing genetic testing.

For the group of parents whose children did not undergo genetic evaluation, reasons included unaware (14%), not interested (20%), cost (20%), time (7%), fearful of results (6%), and never offered (33%) (see Figure 1). Those who were not interested reported reasons such as: already know the cause, not available, doctor mentioned it was unlikely due to genetics, and no plans to have other children. Seventeen percent of parents also indicated other reasons for not considering genetic testing, including concerns about security of DNA samples, difficulty getting an appointment with a genetic testing office, and waiting until child is older.

#### 3.5.2 Genetic testing group

Surprisingly, 11.6% of families who underwent testing did not receive the results. For those who did obtain genetic testing for their child, 52.5% reported that the cause of their child's hearing loss was identified, 42.6% reported it was not identified, and 4.9% were unsure of the results. Genetic specialists were most often the ones to discuss results with the family (26%), followed by genetics counselor (19.2%), ENT physician (6.2%), audiologist (2.1%), pediatrician/family doctor (0.7%), research study personnel (0.7%), and 0.7% of parents did not recall who discussed the results. Less than half of families reported using the results to make decisions about family planning (39.7%) and most were willing to share results of genetic testing with their child (81.1%); 8.1% did not plan on sharing, and 10.8% were unsure. Additionally, most parents (90%) shared their child's results with relatives. Open‐ended responses to why parents did share the results included: awareness, family concern, and family planning while reasons for not sharing included: it was inconclusive, lack of family interest, and personal information.

Surprisingly, for those who underwent genetic testing, over half of parents reported that they did not receive counseling before (55.1%) and only 41.7% reported they received counseling after the testing. However, when they did receive counseling before or after genetic evaluation, they reported that it was effective (71% before testing; 80% after testing), with a parent preference of receiving the results in‐person (65.2%). Furthermore, our data suggest that parents overall continue to be confused with the results regarding the variant of uncertain or unknown significance (VUS). Specifically, 18.3% reported they were Very Confused, 28.3% Somewhat Confused, 20% A Little Confused, and only 33.3% reported Not Confused at All about the VUS. Finally, less than half of the parents (43.4%) remembered what they were told about the mode of inheritance, suggesting that improvements are needed during counseling of genetic results.

## 4 DISCUSSION/CONCLUSION

Given advances in genetic screening and evidence to support its benefits, the current study examined parent perceptions of genetic testing in a sample of pediatric deaf or hard of hearing individuals. The results of an electronic survey found that over 20% of parents continue to be unaware or never offered genetic testing. These findings remain consistent when examining a subtest of parents with children recruited from an otology/ audiology practice in which a geneticist was part of the team. With data supporting that certain genotypes predict progressive hearing loss and others consistently showing better speech perception outcomes, more work is needed to improve the referral rate for genetic testing. Quality improvement projects are warranted to identify the root causes for lack of undergoing testing even when referrals are made.

Other barriers reported by parents are time, cost, and difficulties with scheduling appointments. These barriers are consistent with prior research, suggesting that little has been done to improve the utilization of genetic testing in otolaryngology practices (Lesperance et al., 2018). The literature has reported that both extrinsic and intrinsic reasons likely affect an individual's motivation for pursuing a genetic evaluation. The most commonly reported extrinsic barriers are: receiving a referral, scheduling, or financial concerns regarding the family's insurance. On the other hand, intrinsic reasons may include families not seeing the genetic evaluation as a priority due to dealing with the initial diagnosis and/or intervention options (Lesperance et al., 2018). Our data support these earlier findings, suggesting that intrinsic reasons may be as important as extrinsic reasons when disentangling why families choose to pursue genetic testing or not. The current study also identified similar barriers, including financial/ insurance, difficulty scheduling an evaluation, lack of knowledge, and lack of referral. Interestingly, insurance coverage did not appear to influence a family's decision to undergo testing. In fact, similar rates in completion of genetic testing were found for those with or without coverage. This suggests that other extrinsic or intrinsic factors may be the primary drivers to successfully undergoing a comprehensive genetic evaluation. Improvements in pre‐counseling and education on the benefits of genetic testing is needed universally for individuals with hearing loss and their families, as well as the greater medical community.

Over the past decade, there has been discussion of the importance of adopting precision medicine for hearing loss; however, these benefits will not be seen clinically if adoption rates for genetic testing do not improve (Rudman et al., 2018). Precision medicine or personalized medicine allows for tailoring treatment as it relates to an individual's characteristics, such as genetics, environment, and lifestyle (Zhang, 2015). With the use of next‐generation sequencing (NGS) and expansion of newborn hearing and genetic screening panels, the one‐size fits all approach to medicine will likely diminish with personalized medicine becoming the new standard of care. However, the uptake of personalized medicine is not fully possible until insurance coverage improves allowing equal access to underserved populations, as well as those with state or government insurance plans. In the past, single gene testing was more likely to be covered while NGS testing was rarely covered (Funamura, 2017). Fortunately, with the decreased costs of multigene hearing loss panels, more patients are able to have comprehensive genetic testing covered by insurance (Moon et al., 2023). Thus, it may be the case that individuals whose insurance did not previously cover testing are now covered. As suggested by Li et al. (2022), referral to genetic testing should be considered even outside of the initial diagnosis window as identifying etiology of hearing at any given point may be helpful. Researchers and clinicians need to continue providing evidence of the benefits of incorporating genomic medicine in hearing loss management and treatment to drive insurance companies to improve their coverage for comprehensive genetic testing.

Moreover, patients and families also need to be educated on the overarching benefits of genetic testing. Our study showed that one of the main reasons why parents sought genetic testing was for family planning. A limited number of parents self‐reported pursing genetic testing for treatment planning or prognosis purposes. This highlights a lack of awareness of the overall benefits of genetic testing. In this era of precision medicine, the clinical benefits in terms of clinical management have been well‐documented, including the known relationships between specific genes and progressive hearing loss, and the probability of response to intervention, such as cochlear implantation (Brodie et al., 2023). A study by Brodie et al. (2023) indicated that those who had a genetic diagnosis were 4.6 times more likely to undergo intervention when compared to patients that had a negative or inconclusive result from testing. Thus, highlighting the strong influence that genetic testing may have on a family moving forward with intervention or rehabilitation. These data further support the existing literature that provides strong evidence on the utility of genomic medicine in assisting with determining prognosis, as well as identifying genotypes that are linked with progressive hearing loss, better speech and language outcomes, or comorbidity with other medical conditions that would require evaluation by other medical subspecialties (Carlson et al., 2023).

Our study also identified major gaps in the pre‐ and post‐counseling stages of the genetic evaluation. While the majority of families who underwent genetic testing reported a positive outcome, 12% reported not receiving the results and over half reported not receiving genetic counseling prior. Consistent with prior research our study showed that those parents who received genetic counseling pre‐ or post‐testing reported a more positive experience with counseling and the genetic process. Moreover, those parents who received counseling reported a better understanding of the results. Prior research has also documented that parents of deaf children who receive genetic counseling pre‐ or post‐testing have stronger beliefs regarding genetic origins of hearing loss and have a larger inclination to pursue testing (Withrow et al., 2009). Thus, our data as well as prior work suggest that when provided, genetic counseling is effective; however, there continues to be variability in whether genetic counseling is being provided to this population. Further investigation is warranted to identify the inconsistencies in pre‐ and post‐ counseling services. While the reasons may be related to lack of sufficient providers for this area or limited availability for scheduling, innovative strategies (e.g., use of telemedicine, extended hours, training of students on the field of genetics to increase interest to pursue career in this area) are needed to remediate these barriers.

According to ACMG, regardless of the genetic testing results (positive, negative, inconclusive), they should be communicated via genetic counseling (Li et al., 2022). In fact, they also recommend that for those cases in which no underlying cause was identified, individuals should continue to have follow‐up every 3 years with a geneticist. This is due to changes in genetic testing or test interpretation, symptoms that might not appear in early childhood that are associated with syndromic hearing loss, or identification of medical concerns that warrant a referral to another specialist.

### 4.1 Limitations

While this study provides valuable insights into parent perceptions regarding genetic testing, it also had several limitations. First, this study was conducted in a convenient sample of parents who voluntarily completed the online survey. Thus, respondents had higher education than the general population. Further, our sample was primarily made up of parents who were recruited through social media. While there is evidence to support recruitment of participants via social media for hard‐to‐reach populations (Topolovec‐Vranic & Natarajan, 2016), it is possible that our social media participants are more engaged and thus may have different views about genetic testing compared to the larger general population. Future studies would benefit from multisite studies that will not only assess perceptions, but also address barriers to care. In addition, while our sample was representative of minority individuals in the United States, the majority of respondents were characterized as White. Next, this was a prospective study in which individuals completed the electronic questionnaire after they had already decided or not to pursue genetic testing. Future work should consider quality improvement projects that follow families who are in the process of deciding and/or undergoing testing in order to better capture the influence of timing, referrals, insurance, and scheduling delays on proceeding with testing. This prospective design may also assist in determining the effectiveness of genetic counseling and specific areas that can be improved in the process. Lastly, given that there are no standardized measures to assess parent perceptions in this population, the PPGQT was developed and tailored to the research goals of the study.

### 4.2 Conclusion

In conclusion, genetic testing has shown the highest diagnostic rate of any test for the etiology of childhood deafness and is recommended as one of the first evaluations for children with bilateral sensorineural hearing loss (Shearer & Smith, 2015). Our study highlighted gaps in access to high quality genetic testing, as well as limitations in family knowledge related to the benefits of genetic testing. Genetic testing should be incorporated into the standard of care treatment for patients with pediatric hearing loss; costs for these services need to decrease, and insurance coverage needs to improve. However, our data also revealed that these extrinsic reasons are not the sole reasons for families not pursuing genetic testing. There is a great need to improve education of this patient population related to the benefits of genetic testing so that families can begin to prioritize this as an important diagnostic step in the management of their child's hearing loss. Finally, hearing healthcare professionals and geneticists need to work collaboratively to improve clinical efficiency in serving these patients and families by reducing wait time for scheduling and results as well as providing adequate pre‐ and post‐counseling. Together, genetic testing with newborn hearing screening can continue to improve the identification of hearing loss, as well as reduce time to intervention that is associated with long‐term outcomes in pediatric hearing loss.

## AUTHOR CONTRIBUTIONS

Authors Ivette Cejas, Jennifer Coto, Susan Blanton, Christina M. Sarangoulis, Valerie Yunis, and Xue Zhong Liu confirm that they had full access to all the data in the study and take responsibility for the integrity of the data and the accuracy of the data analysis. All of the authors gave final approval of this version to be published and agree to be accountable for all aspects of the work in ensuring that questions related to the accuracy or integrity of any part of the work are appropriately investigated and resolved. Ivette Cejas, Susan Blanton, Xue Zhong Liu: design of study, design of study questionnaire, and writing of the manuscript; Jennifer Coto: data analysis and writing of the manuscript; Christina M. Sarangoulis: data collection and writing of the manuscript; Valerie Yunis: writing of the manuscript.

## CONFLICT OF INTEREST STATEMENT

Cejas is on the Scientific Program Committee for ACI. She is also on the Board of Directors for AG Bell. She is a consultant for Decibels, Inc. and has received speaker honorariums for ASHA & AG Bell. Authors Coto, Blanton, Yunis, Liu, and Sarangoulis have no disclosures to report.

## ETHICS STATEMENT

Human studies and informed consent: The study was approved by the University of Miami Institutional Review Board. Informed consent was obtained prior to completion of the electronic survey and/or interviews. All surveys were anonymous and data were stored without any identifying information. All procedures followed were in accordance with US Federal Policy for the Protection of Human Subjects.

Animal studies: No non‐human animal studies were carried out by the authors for this article.

## Supporting information


Appendix S1

## Data Availability

The data that support the findings of this study are available from the corresponding author upon reasonable request.
