# Supplementary material for: Parent experiences with genetic testing for pediatric hearing loss
Source: J Genet Couns. 2024 Nov 5;34(3):e1986. doi: 10.1002/jgc4.1986 (PMC12041830; doi:10.1002/jgc4.1986)
Supplement: Supplementary file 1 — Appendix S1 [file JGC4-34-0-s001.pdf]

Name:

Date:

# Parent Perception of Genetic Testing Questionnaire

## Background

1. What kind of health insurance does your child have?

   - ○ Private: through my employer or that of my spouse, partner, or parent, such as an HMO or PPO
   - ○ Private: individually purchased
   - ○ Public: such as Medicaid, Medicare, Social Security Insurance (SSI), or state-sponsored
   - ○ Military, such as Tri-care
   - ○ Self-pay or no insurance
   - ○ Unknown
   - ○ Other (please specify): ______________________________

2. Does your child's health insurance plan include any coverage for genetic testing?

   - ○ Yes
   - ○ No
   - ○ I don't know

3. Please describe your health insurance coverage for genetic testing.

   ________________________________________________________________________________

   ________________________________________________________________________________

4. Please check whether you (the parent) are deaf, hard of hearing or hearing

   - ○ Deaf
   - ○ Hard of hearing (HOH)
   - ○ Hearing

5. If you (the parent) are deaf or HOH, please give the cause of your deafness, if known.

   - ○ Probably genetic (hereditary)
   - ○ Noise damage
   - ○ Infectious disease (rubella, meningitis, CMV)
   - ○ Not sure or unknown
   - ○ Other: _____________

6. What is your (the parent) preferred language?
   - ○ Sign language (ASL, signed English, signed Spanish, etc.)
   - ○ Spoken English
   - ○ Spoken Spanish
   - ○ Other: ________

7. Please say if you (the parent) are more culturally involved with the deaf or hearing community (Check one box)
   - ○ Deaf community
   - ○ Hearing community
   - ○ Equal involvement in both communities
   - ○ Other: _________

8. How would you describe the onset of hearing loss in your child's **left** ear?
   - ○ Sudden (hearing normal before)
   - ○ Progressive (gradually got worse
   - ○ Congenital (since birth)
   - ○ Not Applicable

9. How would you describe the onset of hearing loss in your child's **right** ear?
   - ○ Sudden (hearing normal before)
   - ○ Progressive (gradually got worse
   - ○ Congenital (since birth)
   - ○ Not Applicable

10. Child's age at onset of initial hearing loss: _______________

11. Child's age when hearing loss was confirmed: ______________

12. Please give the cause of your child's hearing loss, if known.
   - ○ Probably genetic (hereditary)
   - ○ Noise damage
   - ○ Infectious disease (rubella, meningitis, CMV)
   - ○ Not sure or unknown
   - ○ Other: _____________

13. Please check what level of hearing loss your child has in his/her left ear.

- ○ Mild (26-40 dB)
- ○ Moderate (41-55 dB)
- ○ Moderately severe (56-70 dB)
- ○ Severe (71-90 dB)
- ○ Profound (91+ dB)
- ○ No hearing loss

14. Please check what level of hearing loss your child has in his/her right ear.

- ○ Mild (26-40 dB)
- ○ Moderate (41-55 dB)
- ○ Moderately severe (56-70 dB)
- ○ Severe (71-90 dB)
- ○ Profound (91+ dB)
- ○ No hearing loss

15. What device(s) does your child use to assist with hearing? (check all that apply)

- ○ Unaided
- ○ Hearing Aid
- ○ BAHA
- ○ Cochlear Implant

16. What is your child's preferred language?

- ○ Sign language (ASL, signed English, signed Spanish, etc.)
- ○ Spoken English
- ○ Spoken Spanish
- ○ Other: __________

17. Please say if your child is more culturally involved with the deaf or hearing community (Check one box)

- ○ Deaf community
- ○ Hearing community
- ○ Equal involvement in both communities
- ○ Other: ______________

## Genetic Testing

There have been many recent advances in the identification of genes which cause deafness. At least half of deaf or hard of hearing people have a genetic cause due to an alteration in one of these genes. Genetic testing for deafness is now available, both for individuals who want to know the cause of their hearing loss, and for family members of deaf or hard of hearing individuals who want to know their chance of having a deaf child. Genetic testing for deafness involves gathering a blood sample from a person and looking at their DNA. DNA is contained in genes, which control the physical characteristics of every person.

18. Which of the following words describes how you feel about new discoveries in the genetics of hearing? (Check all that apply)
    - ○ Excited
    - ○ Concerned
    - ○ Mixed feelings
    - ○ Worried
    - ○ Enthusiastic
    - ○ Horrified
    - ○ Hopeful
    - ○ Positive
    - ○ Cautious
    - ○ Not bothered
    - ○ Confused
    - ○ Negative

19. Whom would you prefer to talk to about the results of genetic testing? (Check all that apply)
    - ○ Geneticist
    - ○ Genetic counselor
    - ○ Audiologist
    - ○ ENT Physician
    - ○ Pediatrician/Family Doctor

20. How important is it for you to have someone who is fluent in your language (not an interpreter) discuss the results of genetic testing?
    - ○ Very important
    - ○ Somewhat important
    - ○ Neutral
    - ○ Not important

21. Has your child had genetic testing for deafness?
    - ○ Yes
    - ○ No (**Skip to question 38**)
    - ○ Unsure (**Skip to question 38**)

---

22. Did you receive the results for your child's genetic testing?
    - ○ Yes
    - ○ No (**Skip to question 30**)

23. Was the cause of your child's hearing loss identified?

- ○ Yes
- ○ No
- ○ Unsure

24. Do you remember the name of the gene?

- ○ Yes
- ○ No

25. What was the name of the gene?

- ○ GJB2: Connexin
- ○ SLC26A4
- ○ MYO15A: Myosin XVA
- ○ OTOF: Otoferlin
- ○ CDH23: Cadherin Related 23
- ○ TMC1: Transmembrane Channel Like 1
- ○ WFS1: Wolframin ER Transmembrane Glycoprotein,
- ○ MYO7A: Myosin VIIA
- ○ Other: ________________

26. Who discussed the results of the testing with you? (Check all that apply)

- ○ Genetics doctor
- ○ Genetic counselor
- ○ Audiologist
- ○ ENT Physician
- ○ Pediatrician/Family Doctor
- ○ Don't remember
- ○ Other

27. Did you share your child's results with relatives? Why or Why Not?

- ○ Yes: ________________________________________________________________

  ________________________________________________________________

  ________________________________________________________________

- ○ No: ________________________________________________________________

  ________________________________________________________________

  ________________________________________________________________

28. Did you use the results to make decisions about family planning?

   ○ Yes

   ○ No

29. In what ways did you use the results to make decisions about family planning?

   ______________________________________________________________________

   ______________________________________________________________________

30. Did you receive counseling before you had genetic testing?

   ○ Yes

   ○ No (**Skip to question 32**)

31. If you received counseling before you had genetic testing, did you find the counseling effective?

   ○ Yes

   ○ No

32. Did you receive counseling after you had genetic testing?

   ○ Yes

   ○ No (**Skip to question 34**)

33. If you received counseling after you had genetic testing, did you find the counseling effective?

   ○ Yes

   ○ No

34. How confused were you regarding variant of uncertain- or unknown- significance (VUS)?

   ○ Very confused

   ○ Somewhat confused

   ○ A little confused

   ○ Not confused at all

35. Do you remember what you were told about mode of inheritance?

- ○ Yes
- ○ No (**Please skip to question 37**)

36. If you remember what you were told about mode of inheritance, please write what you remember about mode of inheritance.

________________________________________

________________________________________

37. What method was the best to help you understand the information you received?

- ○ Face to face interaction
- ○ Written summary
- ○ Other: ____________________

Question 38 and 39 are for parents of children who did not receive genetic testing. If your child received genetic testing **please skip to the Newborn Screening Section (Question 40)**.

---

38. Why have you not considered genetic testing for your child? (Check all that apply)

- ○ Unaware
- ○ Not interested
- ○ Cost
- ○ Time
- ○ Fearful of results
- ○ Other (please explain): ____________________

39. How interested would you be in having genetic testing for your child now?

- ○ Very interested
- ○ Somewhat interested
- ○ Not at all interested

---

## Newborn Screening

You may have heard a lot about Newborn Hearing Screening recently. This refers to screening all babies for hearing loss early in life. It is believed by some that early identification can help improve outcome for deaf children.

40. Which one of these statements best reflects your feelings about newborn hearing screening in general?
    - ○ "I think determining the hearing status of an infant at birth can help parents seek appropriate services right away.''
    - ○ 'I think determining the hearing status of an infant at birth would have no effect on initial care for the infant.''
    - ○ 'I think determining the hearing status of an infant at birth would subject the infant to unnecessary testing and/or procedures.''
    - ○ 'I have not thought about newborn hearing screening.''

As with other newborn screening tests, a follow-up test is required for a diagnosis to be made. Though there are other hearing tests that can be done, genetic testing could also be done.

41. Do you think the most common genes that cause deafness should be routinely tested for in newborn babies? Why or Why Not?
    - ○ Yes: ____________________________________________

      ____________________________________________
    - ○ No: ____________________________________________

      ____________________________________________
    - ○ Unsure: ____________________________________________

      ____________________________________________

**Family Planning**

Information from genetic testing can be used for many purposes. Such purposes include treatment and management options, as well as using the information to make personal decisions.

42. Did you use the results to make decisions about family planning?
    - ○ Very
    - ○ Somewhat
    - ○ Not at all
43. Do you plan on sharing results of genetic testing with your child to assist them in future family planning?
    - ○ Yes
    - ○ No
    - ○ I don't know
44. Would you select a partner so that you could have children of similar or different hearing status than your own? Please explain.
    - ○ Yes: ______________________________________________
      ______________________________________________
    - ○ No: ______________________________________________
      ______________________________________________
    - ○ I don't know: ______________________________________
      ______________________________________________
